# Primary Peritoneal Carcinosarcoma With Metastasis to the Umbilicus and Pancreas

**DOI:** 10.7759/cureus.21309

**Published:** 2022-01-17

**Authors:** Shohei Tanabe, Sachiyo Sugino, Kotaro Ichida, Kiyoshi Niiya, Syuji Morishima

**Affiliations:** 1 Obstetrics and Gynecology, Kobe City Medical Center West Hospital, Kobe, JPN

**Keywords:** peritoneal carcinoma, umbilicus, rare cancer, pancreatic neoplasms, carcinosarcoma

## Abstract

A 51-year-old woman visited our hospital after being diagnosed with ascites effusion by her previous physician due to weight gain for 6 months. Ascites cytology showed adenocarcinoma, MRI showed an omental cake, and CT showed neoplastic lesions in the umbilicus and pancreas. Laparoscopy revealed that the omentum had been replaced by a tumor. Biopsies of the omentum and umbilicus revealed a carcinosarcoma. Treatment with paclitaxel and carboplatin was unsuccessful, and the patient’s general condition deteriorated, leading to her demise. Pathological autopsy revealed carcinosarcoma of peritoneal origin metastasizing to the umbilicus and tail of the pancreas. No tumors were found in the uterus, ovaries, or fallopian tubes.

## Introduction

Primary peritoneal carcinosarcoma is an extremely rare disease, with only 40 cases reported in the literature. It is most common in women over the age of 40 and progresses very quickly [1]. We report the first case of primary peritoneal carcinosarcoma metastasizing to the umbilicus and pancreas and present a review of the literature. This report was approved by the Clinical Research Review Committee of our hospital after obtaining written informed consent from the patient and her family.

## Case presentation

A 51-year-old nulliparous, nulligravid woman with a history of rheumatoid arthritis for which she was taking a tumor necrosis factor (TNF)-α inhibitor was referred to our hospital for ascites and suspicion of carcinomatous peritonitis. According to the patient, she had been “feeling fat” for approximately 6 months before visiting her family doctor.

**Figure 1:**
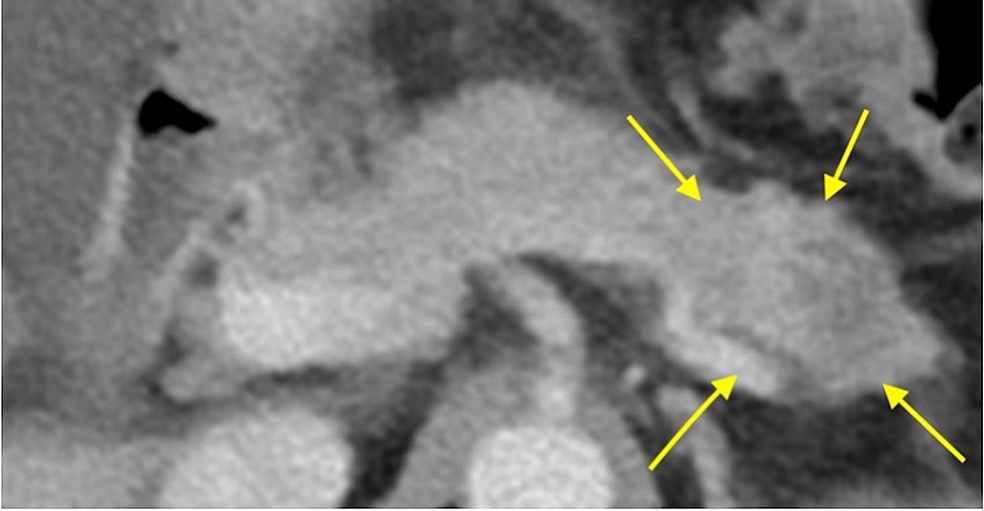
Computed tomography of the pancreas The tumor (yellow arrows) is located in the tail of the pancreas.

**Figure 2:**
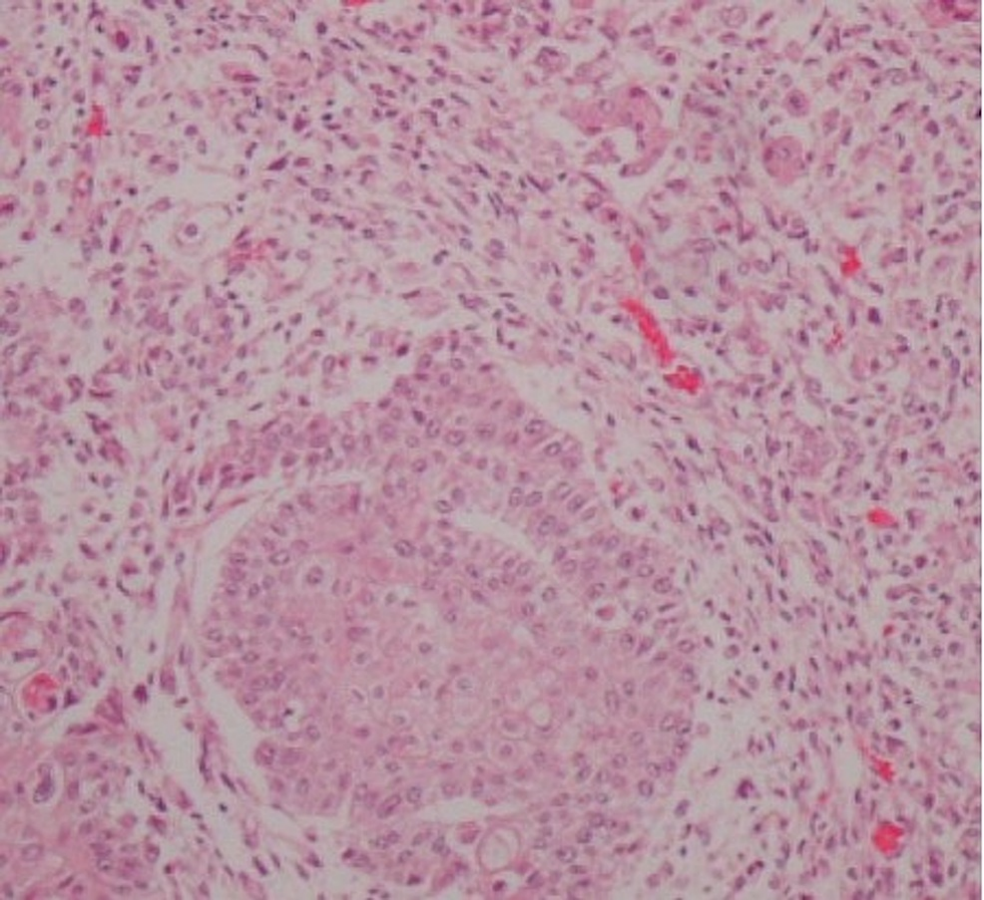
Biopsy of the omentum The tumor cells contain a mixture of epithelial and stromal components with glandular tubular structures (hematoxylin and eosin stain ×100).

**Figure 3:**
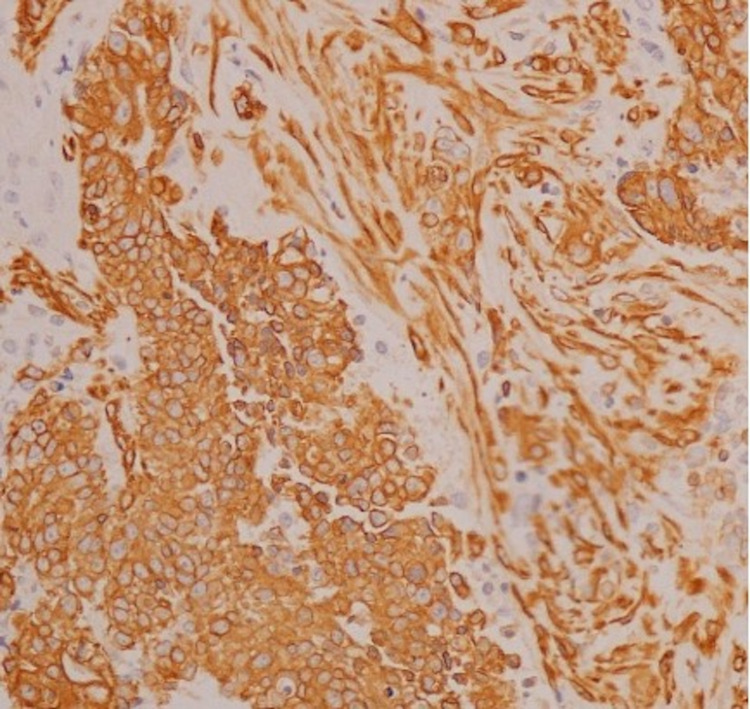
Biopsy of the omentum Cytokeratin AE1/AE3, which stains epithelial cell-derived tumor cells, is generally positive. Therefore, tumor cells in the stromal component are also of epithelial cell origin (cytokeratin AE1/AE3 ×100).

**Figure 4:**
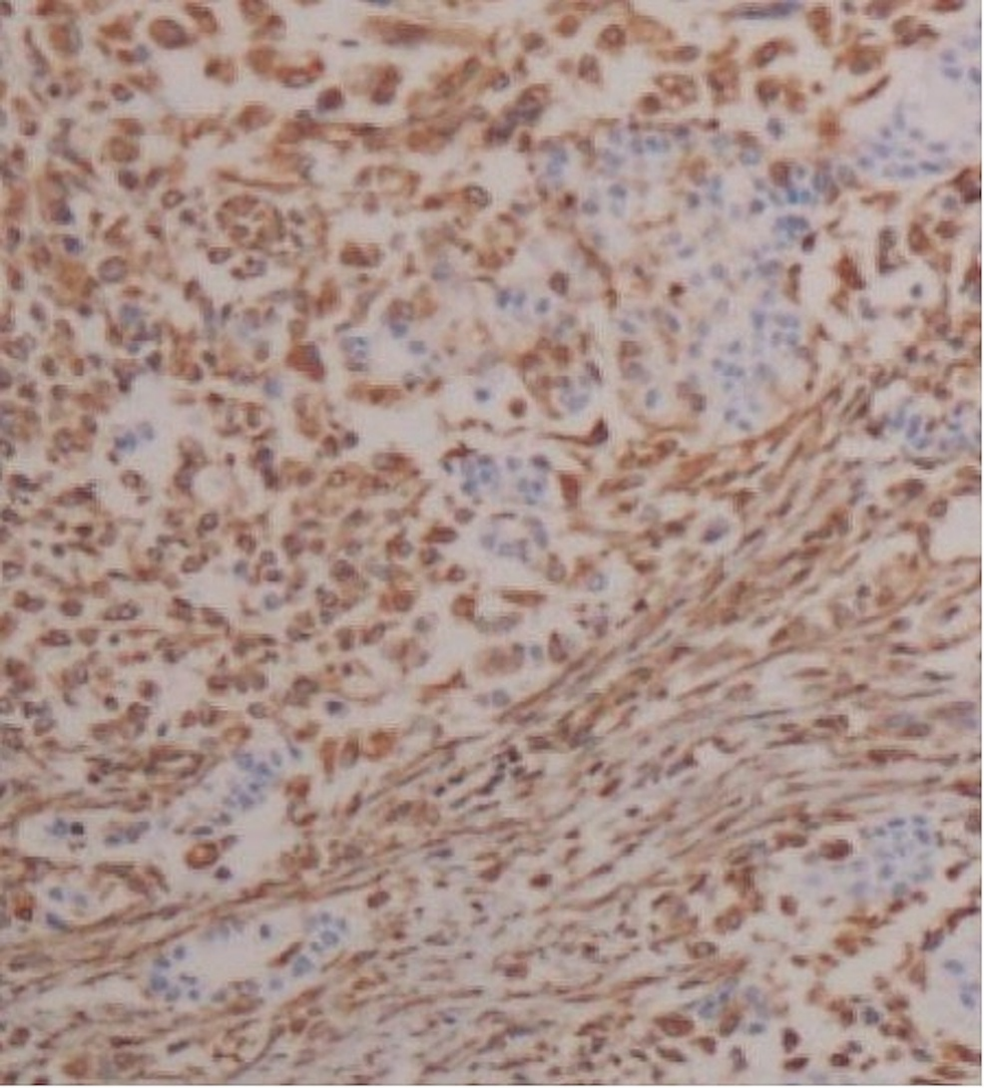
Biopsy of the omentum Vimentin, which stains tumor cells of epithelial cell origin, is generally positive. Therefore, tumor cells in the stromal component are also of epithelial cell origin (vimentin ×100).

A transabdominal ultrasonographic examination revealed ascitic fluid accumulation in the entire peritoneal cavity. Additionally, granulation tissue was found in the umbilicus. Ascitic tap yielded 1,200 mL of fluid, which showed adenocarcinoma of cytological examination. MRI showed an omental cake, and CT showed a suspicious mass in the tail of the pancreas (Figure 1). Laparoscopic surgery was performed, and intraoperative findings showed no gross lesions in the uterus, ovaries, or intestinal tract. However, the omentum was entirely replaced by a tumorous mass. Biopsies of the omentum and peritoneum were taken. Histologically, hematoxylin and eosin staining showed an adenocarcinoma with a sarcomatous component. Immunohistochemically, both components were positive for cytokeratin AE1/AE3 and vimentin (Figures 2-4). A biopsy of the umbilical lesion revealed a carcinosarcoma, similar to that of the omentum. Biopsies of the ovaries and fallopian tubes showed no pathological lesions. As the largest lesion was found in the omentum, a diagnosis of multiple metastases of primary peritoneal carcinosarcoma was reached. The patient’s aunt had died of breast cancer at the age of 40; therefore, genetic testing for BReast CAncer gene (BRCA) mutations was performed; however, none were found.

**Figure 5:**
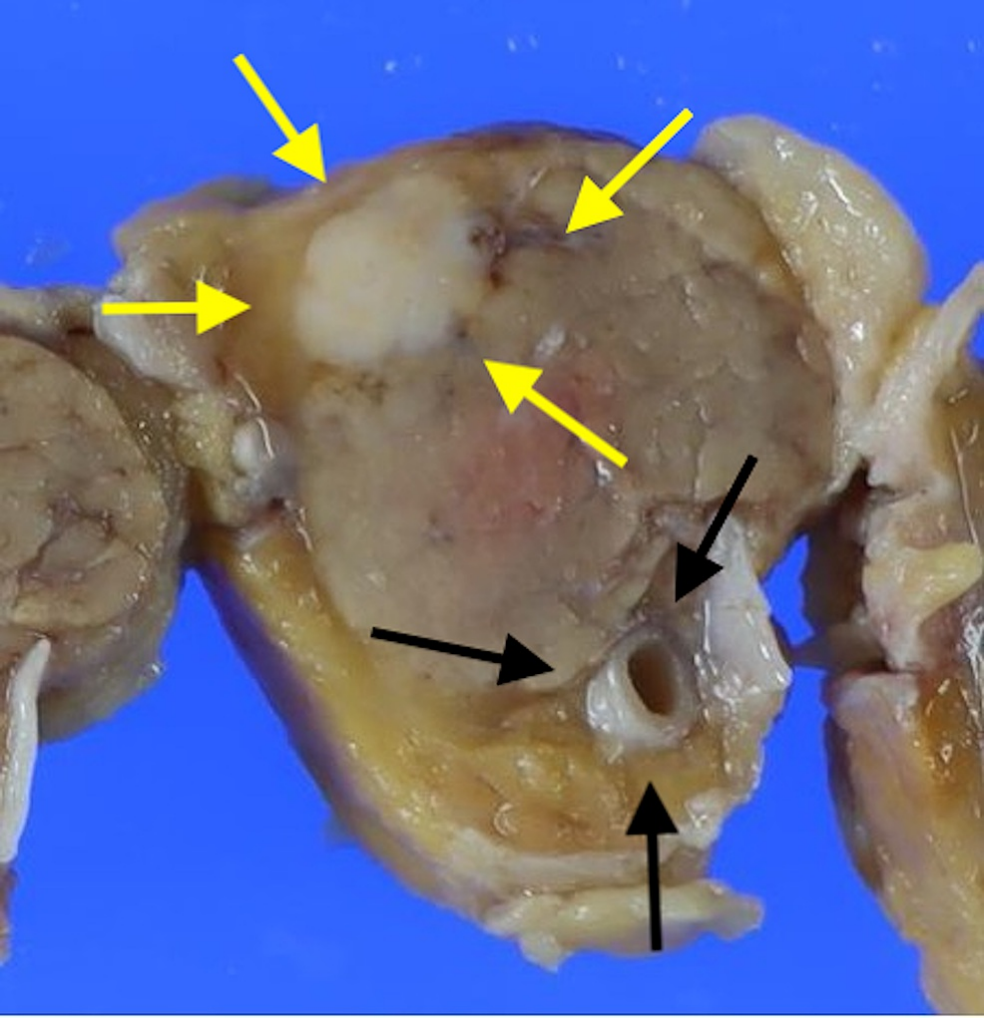
The tumor (yellow arrows) is located close to the membrane It is separated from the pancreatic duct (black arrows) and is not a typical image of pancreatic cancer. Peritoneal carcinoma dissemination occurred through the membrane.

Paclitaxel and carboplatin (TC) therapy was initiated, which was unsuccessful; thereafter, the patient was admitted for intestinal obstruction and underwent reoperation. Intraoperative findings showed that the tumor had progressed and invaded the entire abdominal cavity, including the small intestine, causing intestinal obstruction. Partial resection of the small intestine was performed to relieve the intestinal obstruction, and total hysterectomy with bilateral adnexectomy was performed for debulking surgery of peritoneal cancer. At the end of the operation, the patient’s blood pressure dropped, and she was admitted to the intensive care unit. Her general condition deteriorated further, and she died of the primary disease on the 51st day after she visited our hospital. The postmortem pathological autopsy revealed a neoplastic lesion in the tail of the pancreas. Unlike the findings of pancreatic cancer originating from the pancreatic duct, the tumor had invaded from the pancreatic surface toward the center of the pancreas (Figure 5). The lesion in the tail of the pancreas showed carcinosarcoma features, similar to that in the omentum. No tumors were found in the uterus, ovaries, or fallopian tubes. Therefore, the patient was diagnosed with advanced (stage 4B) primary peritoneal carcinosarcoma with multiple metastases to the umbilicus and pancreas.

## Discussion

Here, we report the first known case of primary peritoneal carcinosarcoma metastasizing to the umbilicus and pancreas in a 51-year-old female who was not successfully treated. Carcinosarcomas arising in the female reproductive organs are classified as malignant mixed Müllerian tumors (MMMT). Carcinosarcomas originating from the peritoneum are extremely rare and associated with a poor prognosis due to rapid progression [2]. In this case, a primary carcinosarcoma of the omentum was diagnosed after it had grown large enough to cause symptoms. The diagnosis was largely based on imaging [3]. Currently, there is no consensus on treatment, and there are only a few prospective studies on carcinosarcoma [4]. In previous retrospective studies, the goal of early surgery was complete resection, and that of advanced surgery was tumor reduction. Platinum-based chemotherapy is used as adjuvant chemotherapy [5].

As primary peritoneal carcinosarcoma is extremely rare, a case analysis of MMMT has been reported, which includes cases of primary ovarian and fallopian tube carcinosarcoma [6]. In the present case, pancreatic tail resection was indicated but could not be performed because of its high invasiveness. A case series of ovarian carcinosarcoma reported that patients who underwent optimal surgery with a residual tumor less than 2 cm survived longer [7]. However, even if optimal surgery had been performed in this case, it is questionable whether the prognosis would have improved. Patients with advanced ovarian carcinosarcoma respond more poorly to platinum/taxane-based anticancer drugs than those with serous ovarian cancer [7]. In the present case, the patient had advanced cancer with distant metastasis at presentation, and it is doubtful whether the prognosis would have improved even if the lesion had been completely resected in the first surgery.

Pancreatic metastasis from ovarian cancer is rare, with only 17 reported cases. Therefore, when treating pancreatic tumors, it is critical to determine whether the tumor is a primary pancreatic cancer or a pancreatic metastasis of a different primary tumor [8]. In the present case, as the main lesion was found in the omentum on laparoscopic examination and the pancreatic tail lesion was considered too small to be the source of distant metastasis, metastatic peritoneal cancer was diagnosed. A metastatic lesion in the umbilicus is called a Sister Mary Joseph nodule, which is rare, accounting for 1-3% of neoplasms in the abdominal and pelvic regions, and is associated with poor prognosis [9]. These lesions are usually adenocarcinomas [10], and only one case of carcinosarcoma metastasizing to the umbilicus has been reported [11].

## Conclusions

The present case is the first of primary peritoneal carcinosarcoma metastasizing to the umbilicus and pancreas. It was refractory to anticancer drugs and progressed rapidly. In this case, we had trouble differentiating the tumor from pancreatic cancer because it was located in the tail of the pancreas, which is difficult to biopsy. The patient was rushed to undergo anticancer drug therapy but could not cope with the rapid progression of the cancer. Although carcinosarcoma of peritoneal origin is rare, it is advisable to administer anticancer drugs and perform surgery as soon as possible in suspected cases. In this case, the cancer was already advanced when it was diagnosed, so anticancer drug treatment was given priority. However, if the patient is deemed to be operable at the time of diagnosis, complete removal of cancer by surgery may be considered.
